# Value of texture analysis based on dynamic contrast-enhanced magnetic resonance imaging in preoperative assessment of extramural venous invasion in rectal cancer

**DOI:** 10.1186/s13244-022-01316-2

**Published:** 2022-11-22

**Authors:** Junjie Fang, Wei Sun, Dan Wu, Peipei Pang, Xiuyu Guo, Chunyao Yu, Wei Lu, Guangyu Tang

**Affiliations:** 1 Department of Radiology, Shanghai Tenth People’s Hospital, School of Medicine, Tongji University, No. 301 Yanchang Road, Shanghai, 200072 People’s Republic of China; 2 Department of Radiology, Hwa Mei Hospital, University of Chinese Academy of Sciences, No. 41 Northwest Street, Haishu District, Ningbo, 315010 Zhejiang People’s Republic of China; 3 Key Laboratory of Diagnosis and Treatment of Digestive System Tumors of Zhejiang Province, Ningbo, 315000 People’s Republic of China; 4 Department of Radiology, Sir Run Run Shaw Hospital, Zhejiang University School of Medicine, Hangzhou, 310002 People’s Republic of China; 5 Department of Pharmaceuticals Diagnosis, GE Healthcare, Hangzhou, 310002 People’s Republic of China

**Keywords:** Rectal neoplasms, Magnetic resonance imaging, Neoplasm staging, Machine learning

## Abstract

**Objective:**

Accurate preoperative assessment of extramural vascular invasion (EMVI) is critical for the treatment and prognosis of rectal cancer. The aim of our research was to develop an assessment model by texture analysis for preoperative prediction of EMVI.

**Materials and methods:**

This study enrolled 44 rectal patients as train cohort, 7 patients as validation cohort and 18 patients as test cohort. A total of 236 texture features from DCE MR imaging quantitative parameters were extracted for each patient (59 features of *K*^trans^, *K*_ep_, *V*_e_ and *V*_p_), and key features were selected by least absolute shrinkage and selection operator regression (LASSO). Finally, clinical independent risk factors, conventional MRI assessment, and T-score were incorporated to construct an assessment model using multivariable logistic regression.

**Results:**

The T-score calculated using the 4 selected key features were significantly correlated with EMVI (*p* < 0.010). The area under the receiver operating characteristic curve (AUC) was 0.797 for discriminating between EMVI-positive and EMVI-negative patients with a sensitivity of 88.2% and specificity of 70.4%. The conventional MRI assessment of EMVI had a sensitivity of 23.53% and a specificity of 96.30%. The assessment model showed a greatly improved performance with an AUC of 0.954 (sensitivity, 88.2%; specificity, 92.6%) in train cohort, 0.833 (sensitivity, 66.7%; specificity, 100%) in validation cohort and 0.877 in test cohort, respectively.

**Conclusions:**

The assessment model showed an excellent performance in preoperative assessment of EMVI. It demonstrates strong potential for improving the accuracy of EMVI assessment and provide a reliable basis for individualized treatment decisions.

**Supplementary Information:**

The online version contains supplementary material available at 10.1186/s13244-022-01316-2.

## Key points

- EMVI is a key factor in treatment selection and prognostication of patients with rectal cancer.
- We developed an assessment model by texture analysis based on DCE MRI.
- The assessment model showed an excellent performance in preoperative assessment of EMVI.

## Introduction

Rectal cancer is diagnosed in more than 100,000 cases annually worldwide [1–3]. Extramural vascular invasion (EMVI) is defined as the presence of tumor cells in the vasculature beyond the muscularis propria [4]. It only occurs in at least *T*3-stage tumors, which means locally advanced stage, and is associated with recurrence, metastasis, and poor prognosis [5–7]. Despite no current conclusive data on the prognostic importance of margin involvement by EMVI, the mesorectal fascia (MRF) should be considered as involved in  the case of a margin of ≤ 1 mm from EMVI [8]. Therefore, an accurate preoperative assessment of EMVI plays a critical role in differentiating between *T*2 and *T*3 stage to provide patients individualized treatment for improved survival and quality of life.

Magnetic resonance imaging (MRI) plays an important role in preoperative assessment of tumor staging and circumferential resection margin (CRM) in patients with rectal cancer [9–11] and has a better accuracy than computed tomography (CT) and a better repeatability than endoscopic ultrasound (EUS) [10, 12]. MRI is also useful in preoperatively assessing EMVI [13], but the sensitivity is unstable (28% to 100%) [14–16]. Dynamic contrast-enhanced (DCE) MRI assesses tumor perfusion, which is related to tumor microcirculation. Although DCE sequences are not routinely recommended [17], it has been widely used in the differentiation of benign and malignant tumors, staging, and therapeutic response evaluation in rectal cancer [18–20]. DCE sequences could be an effective supplement to conventional MRI, to improve the preoperative assessment of EMVI in rectal cancer. Texture analysis (TA) is an important part of radiomics and is widely used for computer-aided image assessment [21]. It can provide objective and detailed information about tumor heterogeneity and its microenvironment, which are closely related to staging, response to treatment, and prognosis [22, 23]. Some previous studies showed that texture features were valuable in predicting the efficacy of neoadjuvant chemoradiotherapy (nCRT) for rectal cancer and tumor recurrence [24–26]. TA could be a potential method to improve EMVI assessment by extracting a large amount of information from quantitative parametric maps.

The purpose of this study was to evaluate the efficiency of texture features of DCE MRI parameters, conventional MRI, and clinical information for preoperative assessment of EMVI in patients with rectal cancer that will provide a noninvasive and reliable basis for individualized treatment decisions.

## Materials and methods

### Patients

This retrospective study was approved by the ethics committee of our hospital. The required informed patient consent was waived. We collected patients who underwent surgical treatment from May 2019 to December 2019 for model construction (train cohort), who underwent surgical treatment from January 2020 to August 2020 for validation (validation cohort), and who underwent surgical treatment from September 2020 to July 2021 for test (test cohort). All patients underwent pelvic DCE MRI before surgery.

The inclusion criteria were as follows: (1) primary tumor was histopathologically proven to be rectal cancer; (2) postoperative pathological diagnosis of EMVI was confirmed; (3) preoperative pelvic DCE MRI was performed; and (4) patients had never been treated with neoadjuvant chemoradiotherapy. The exclusion criteria were as follows: (1) primary tumor was not clearly visible on MR images because of artifacts, (2) primary tumor displayed incompletely on DCE images, and (3) other malignancies were present. The pathological EMVI was diagnosed by the consecutive sections of the entire en-bloc specimen when tumor cells were found contacting tightly with endothelial cells in vasculature, including blood and lymph vessels, without specifying the intra- or extra-mural invasion. And then the patients were divided into 2 groups by the pathological EMVI: EMVI-positive (*n* = 17, 3, 5 in train, validation and test cohorts, respectively) and EMVI-negative (*n* = 27, 4, 13 in train, validation and test cohorts, respectively).

We also gathered the following patients’ clinical characteristics: sex, age, body mass index (BMI), carcinoembryonic antigen (CEA), and carbohydrate antigen 19-9 (CA19-9). BMI, an indicator of body fat, is calculated using the individual's weight and height according to the following formula: ${\text{BMI}}\left( {{\text{kg/m}}^{2} } \right) = {\text{Weight}}\left( {{\text{kg}}} \right)/\left( {{\text{Height}}\left( {\text{m}} \right)} \right)^{2}$.

### MRI data acquisition

All patients underwent preoperative pelvic MRI scans in the supine position on a 1.5-Tesla MR scanner (Avanto, Siemens, German) with a phased-array body coil. The scan sequences included *T*_1_WI (transverse position), *T*_2_WI (transverse and sagittal position), high-resolution *T*_2_WI (axial position of abnormal intestinal segment), DWI (*b*-value of 0 and 800 s/mm^2^) and DCE-MRI. The DCE-MRI adopted 3D-volume interpolated examination (3D-VIBE) and the scan parameters were as follows: multiple flip angles of 5°, 10°, 15°, respectively; each angle was scanned one time, each acquisition time was 8 s, total time was 24 s; repetition time (TR) = 4.88 ms; echo time (TE) = 2.39 ms; Average = 1; field-of-view (FOV) = 350 × 280 mm^2^; matrix = 288 × 196; slice thickness = 4.0 mm; and bandwidth (BW) = 400 Hz/px. The DCE parameters were the same as the multi-angle parameter; the flip angle was 10°; multiphase dynamic enhancement scanned 30 phases; the imaging time was 4 min. We injected 0.1 mmol/kg gadolinium diamide (Omniscan; GE Healthcare, Little Chalfont, UK, http: //www. gehealthcare.com) in the center of the elbow using the high-pressure vein syringe and then injected 20 mL of physiological saline at the speed of 2 mL/s. The scan parameters of the other scan sequences were described in the Additional file 1: Appendix A1.

All MR images were retrieved from the picture archiving and communication system (eWorld, China).

### Conventional MRI assessment

Two radiologists with 10 (W.L.) and 15 (J.F.) years of experience in abdominal radiology assessed rectal cancer based on MRI individually. The assessment included the distance between the rectal cancer and anal edge (distance), MRI-based T staging (cT), MRI-based regional lymph node metastasis assessment (cN), number of visible regional lymph nodes on MR images (LN), MRI-based EMVI assessment (MR-EMVI) and MRI-based CRM assessment (MR-CRM). Disagreements were resolved by consensus or consultation with a third radiologist (G.T.) with over 25 years of experience in abdominal radiology. All radiologists were blinded to the histological results.

### Data processing and tumor segmentation

DCE MRI images were transferred to quantitative Omni kinetics software (OK, GE Healthcare, China). First, the arterial input function (AIF) was placed on the proximal abdominal aorta and the AIF curve was obtained. Then, the extended Tofts linear model was selected to obtain the pharmacokinetic parameters *K*^trans^, *K*_ep_, *V*_e_, and *V*_p_ (*K*^trans^: volume transfer constant between the blood plasma and the extracellular extravascular space (EES), *K*_ep_: rate constant of contrast agent escapes from the EES into the plasma compartment, *V*_e_: EES volume fraction, and *V*_p_: plasma volume fraction). Three-dimensional tumor segmentation was performed by two radiologists D.W. and W.S. The region of interest (ROI), covering the whole primary tumor without the adjacent tissue, lumen, or intestinal content, was outlined on the original images first and then copied into the *K*^trans^, *K*_ep_, *V*_e_ and *V*_p_ maps.

The texture features of the above pharmacokinetic parameters were extracted by OK software, including histogram features (number = 12), grey-level co-occurrence matrix (GLCM) features (number = 13), Haralick features (number = 9), grey-level run-length matrix (RLM) features (number = 16), and formfactor features (number = 9). Fifty-nine features were computed using every pharmacokinetic parameter, and a total of 236 features were obtained.

To evaluate the feature reproducibility across different radiologists, 30 cases were randomly selected for a double-blinded comparison of manual segmentations by the two radiologists. The inter-observer agreement of the features was evaluated using the interclass correlation coefficient (ICC). An ICC of > 0.75 was considered as a mark of excellent reliability.

### Texture features selection

To eliminate the differences in the value scales of extraction features, feature normalization was performed before feature selection. Each feature for all patients was standardized using Z-scores.

Analysis of variance (ANOVA) and Kruskal–Wallis tests were performed to select the texture features significantly correlated with EMVI. Then, the least absolute shrinkage and selection operator (LASSO) regression method by penalty parameter tuning *λ* were used to reduce the redundancy or selection bias of the features. Optimal λ was chosen based on the minimum criteria in a tenfold cross-validation. This method is widely used for high-dimensional features with small medical images.

After feature selection, texture score (T-score) was derived from the linear combination of the selected features that were weighted by their respective LASSO coefficients to reflect the probability of EMVI for each patient. The predictive capability was evaluated using the receiver operating characteristic (ROC) curve and area under the curve (AUC).

### Assessment model construction

Univariate logistic regression was used for clinical information and conventional MRI features to select independent clinical predictors. Multivariable logistic regression analysis with the selected independent clinical risk factors and T-score was applied to develop a combined model for the EMVI assessment model. Then, we used the validation cohort to conduct a preliminary assessment of the model.

A backward stepwise multivariable logistic regression was performed using the Akaike information criterion (AIC) as the stop rule. To provide the clinician with a quantitative tool for predicting the individual probability of EMVI, a nomogram was plotted based on the EMVI assessment model. The differences between the ROC curves of MR-EMVI, T-score, and the assessment model were compared using the DeLong test.

Finally, the assessment model was tested by a test cohort.

### Clinical practice

To estimate the incremental utility of the T-score and assessment nomogram model, the decision curve of the different models was plotted for the entire dataset. The decision curve analysis (DCA) informs the patient or doctor which of the several models are optimal using a threshold probability.

### Statistical analysis

All statistical analyses were performed using R software (version 3.5.1; http://www.Rproject.org). Univariate analysis for clinical features was implemented using an independent-sample *t* test or the Mann–Whitney *U* test for continuous variables, and the Chi-squared test for categorical variables. All statistical significance levels were two-sided, with statistical significance set at 0.05. The LASSO logistic regression was conducted using the “glmnet” package in R software. Multivariate logistic regression analysis was performed using the “stats” package. Lastly, nomogram construction was done using the “rms” package.

## Results

### Clinical characteristics and conventional MRI assessments

This study included a total of 69 rectal cancer patients with an EMVI-positive rate of 36.23% (Additional file 1: Figure S1). There were 44 patients (male, *n* = 34; female, *n* = 10; mean age, 64.18 ± 12.97 years; range 36–92 years) in train cohort, 7 patients (male, *n* = 7; female, *n* = 0; mean age, 74.00 ± 9.59 years; range 55–85 years) in validation cohort, and 18 patients (male, *n* = 15; female *n* = 3; mean age, 67.56 ± 11.10 years; range 43–92 years) in test cohort. All patients underwent surgical treatment in 2 weeks after DCE-MRI without nCRT, which was determined by the surgeons’ reassessment and patients’ preference. The associations between EMVI and the risk factors of clinical characteristics and conventional MRI assessments are summarized in Tables 1, 2, and 3. None of the clinical characteristics showed significant association with EMVI. On the other hand, cT, cN, and LN of conventional MRI assessment demonstrated a significant association with EMVI. The EMVI assessed by radiologists based on MRI had a sensitivity of 16.00% and a specificity of 88.64%.

**Table 1:** Associations between EMVI and clinical risk predictors in train cohort

Characteristics	EMVI (−) (*N* = 27)	EMVI (+) (*N* = 17)	*p*
Age, mean ± SD, years	66.11 ± 12.24	61.12 ± 13.89	0.211
Sex, *N* (%)			0.638
Male	22 (81.48)	12 (70.59)	
Female	5 (18.52)	5 (29.41)	
BMI, mean ± SD, kg/m^2^	23.31 ± 2.88	21.84 ± 2.36	0.078
CEA		0.195	
≤ 5 ng/mL, *N* (%)	22 (81.48)	10 (58.82)	
> 5 ng/mL, *N* (%)	5 (18.52)	7 (41.18)	
CA19-9			0.870
≤ 34 U/mL, *N* (%)	24 (88.89)	14 (82.35)	
> 34 U/mL, *N* (%)	3 (11.11)	3 (17.65)	
Distance, mean ± SD, mm	68.85 ± 25.62	81.18 ± 39.87	0.211
cT *N* (%)			0.001
*T*1	1 (3.70)	0 (0)	
*T*2	7 (25.93)	1 (5.88)	
*T*3	17 (62.96)	9 (52.94)	
*T*4a	2 (7.41)	6 (35.29)	
*T*4b	0 (0)	1 (5.88)	
cN *N* (%)			0.002
Negative	23 (85.19)	6 (35.29)	
Positive	4 (14.81)	11 (64.71)	
LN, median, *N*	0	5	< 0.001
MR-EMVI, *N* (%)			0.126
Negative	26 (96.30)	13 (76.47)	
Positive	1 (3.70)	4 (23.53)	
MR-CRM, *N* (%)*			0.675
Negative	26 (96.30)	15 (88.24)	
Positive	1 (3.70)	2 (11.76)	

Associations between EMVI and clinical risk predictors in train cohort

*EMVI* extramural vascular invasion, *SD* standard deviation, *BMI* body mass index, *Distance* the distance between the rectal cancer and anal edge, *cT* MRI-based T staging, *cN* MRI-based N staging, *LN* number of visible regional lymph nodes, *MR-EMVI* MRI-based EMVI assessment, *MR-CRM* MRI-based circumferential resection margin assessment

*Pathological CRM in all patients were negative

**Table 2:** Associations between EMVI and clinical risk predictors in validation cohort

Characteristics	EMVI (−) (*N* = 4)	EMVI (+) (*N* = 3)
Age, mean ± SD, years	77.00 ± 5.48	70.00 ± 13.75
Sex, *N* (%)		
Male	4 (100)	3 (100)
Female	0 (0)	0 (0)
BMI, mean ± SD, kg/m^2^	22.40 ± 1.86	21.12 ± 4.92
CEA		
≤ 5 ng/mL, *N* (%)	4 (100)	1 (33.33)
> 5 ng/mL, *N* (%)	0 (0)	2 (66.67)
CA19-9		
≤ 34 U/mL, *N* (%)	4 (100)	3 (100)
> 34 U/mL, *N* (%)	0 (0)	0 (0)
Distance, mean ± SD, mm	85.00 ± 20.82	99.67 ± 22.14
cT *N* (%)		
*T*1	0 (0)	0 (0)
*T*2	0 (0)	0 (0)
*T*3	3 (75.00)	1 (33.33)
*T*4a	1 (25.00)	2 (66.67)
*T*4b	0 (0)	0 (0)
cN *N* (%)		
Negative	1 (25.00)	1 (33.33)
Positive	3 (75.00)	2 (66.67)
LN, median, N	3.5	3
MR-EMVI, N (%)		
Negative	3 (75.00)	3 (100)
Positive	1 (25.00)	0 (0)
MR-CRM, *N* (%)*		
Negative	4 (100)	3 (100)
Positive	0 (0)	0 (0)

Associations between EMVI and clinical risk predictors in validation cohort

*EMVI* extramural vascular invasion, *SD* standard deviation, *BMI* body mass index, *Distance* the distance between the rectal cancer and anal edge, *cT* MRI-based T staging, *cN* MRI-based N staging, *LN* number of visible regional lymph nodes, *MR-EMVI* MRI-based EMVI assessment, *MR-CRM* MRI-based circumferential resection margin assessment

*Pathological CRM in all patients were negative

**Table 3:** Associations between EMVI and clinical risk predictors in test cohort

Characteristics	EMVI (−) (*N* = 13)	EMVI (+) (*N* = 5)
Age, mean ± SD, years	69.5 ± 9.9	62.6 ± 13.8
Sex, *N* (%)		
Male	11 (84.62)	4 (80)
Female	2 (15.38)	1 (20)
BMI, mean ± SD, kg/m^2^	21.9 ± 1.9	21.6 ± 2.5
CEA		
≤ 5 ng/mL, *N* (%)	6 (46.2)	5 (100)
> 5 ng/mL, *N* (%)	7 (53.8)	0 (0)
CA19-9		
≤ 34 U/mL, *N* (%)	12 (92.3)	5 (100)
> 34 U/mL, *N* (%)	1 (7.7)	0 (0)
Distance, mean ± SD, mm	63.3 ± 23.1	78.2 ± 15.3
cT *N* (%)		
*T*1	0 (0)	0 (0)
*T*2	3 (23.1)	2 (40)
*T*3	7 (53.8)	3 (60)
*T*4a	3 (23.1)	0 (0)
*T*4b	0 (0)	0 (0)
cN *N* (%)		
Negative	2 (15.4)	2 (40)
Positive	11 (84.6)	3 (60)
LN, median, *N*	1	2
MR-EMVI, *N* (%)		
Negative	10 (76.9)	5 (100)
Positive	3 (23.1)	0 (0)
MR-CRM, *N* (%)*		
Negative	13 (100)	5 (100)
Positive	0 (0)	0 (0)

Associations between EMVI and clinical risk predictors in test cohort

*EMVI* extramural vascular invasion, *SD* standard deviation, *BMI* body mass index, *Distance* the distance between the rectal cancer and anal edge; *cT* MRI-based T staging, *cN* MRI-based N staging, *LN* number of visible regional lymph nodes, *MR-EMVI* MRI-based EMVI assessment, *MR-CRM* MRI-based circumferential resection margin assessment

*Pathological CRM in all patients were negative

### Texture analysis

**Fig. 1:**
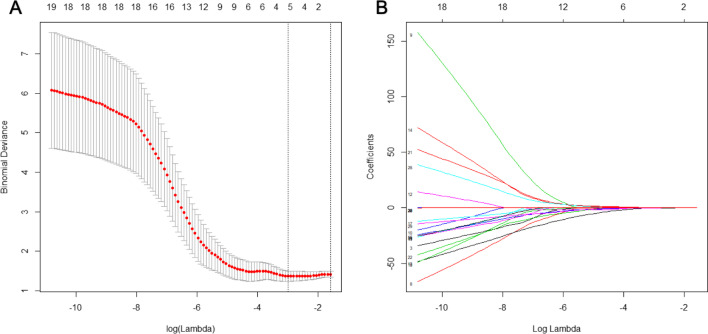
Key texture features selection of DCE MRI quantitative parameters by LASSO analysis. **a** The value of regularization parameter (*λ*) selected by tenfold cross-validation when the deviance was minimal. **b** A coefficient profile plot of 33 texture features was produced against the log (*λ*) sequence. Total 4 nonzero coefficients were selected finally by the optimal λ. LASSO, least absolute shrinkage and selection operation

We extracted a total of 236 texture features from DCE MRI quantitative parameters for each patient (59 features of *K*^trans^, *K*_ep_, *V*_e_, and *V*_p_). The ICC values for all features were greater than 0.75, indicating a good reproducibility. Among the 33 features selected by ANOVA and Kruskal–Wallis test, 4 optimal key features (Additional file 1: Appendix A2) were selected by the LASSO method (Fig. 1) and then constituted T-score. The calculation formula for T-score is as follows:

$$\begin{aligned} T{\text{ - score}} & = - 0.759 - 0.937 \times {\text{Kep}}\_{\text{Correlation}} + 0.351 \times {\text{Kep}}\_{\text{Clustershade}} + 0.504 \\ & \quad \times {\text{Kep}}\_{\text{SumEntropy}} - 0.368 \times {\text{Vp}}\_{\text{HaraVariance}} \\ \end{aligned}$$

**Fig. 2:**
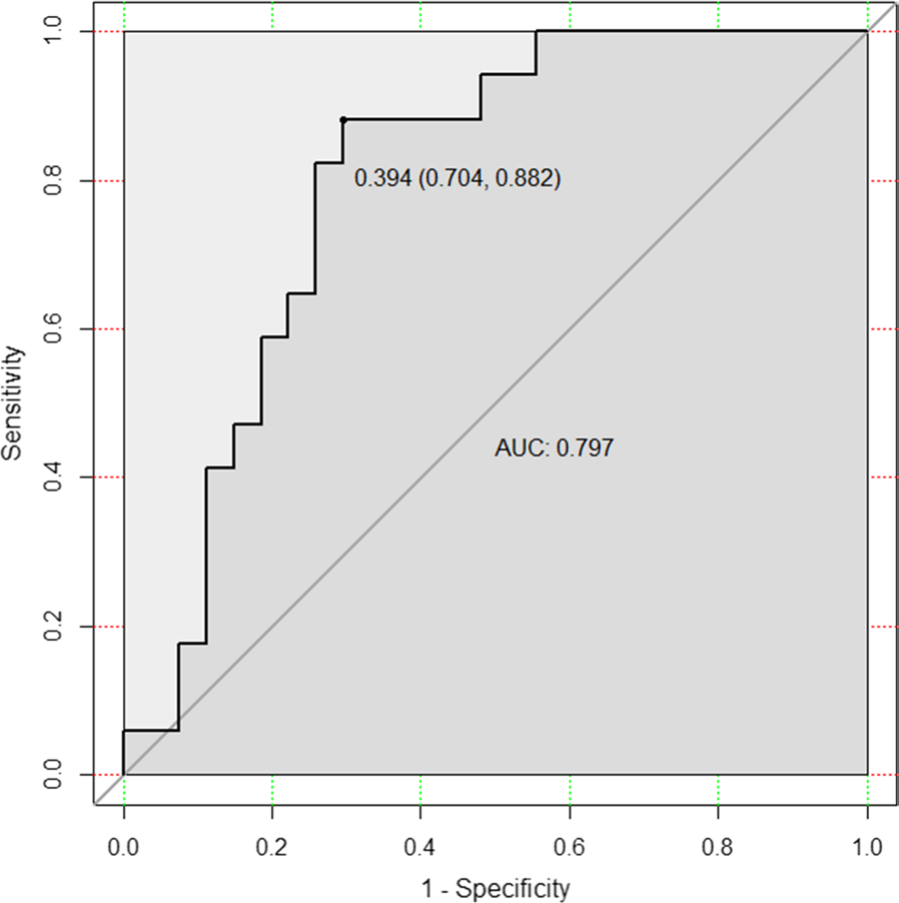
ROC curve of T-score. ROC, receiver operating characteristic; AUC, area under ROC curve; T-score, the texture score calculated by linear combination of the selected features which were weighted by their respective LASSO coefficients. When the cutoff value is 0.394, the specificity is 0.704 and the sensitivity is 0.882

T-score with an AUC of 0.797 (95% confidence interval [CI] 0.704–0.882), sensitivity of 0.882 and specificity of 0.704 (Fig. 2). The DeLong test showed its better performance than MR-EMVI (*Z* = 2.032, *p* = 0.042) in assessing EMVI.

### Assessment model construction

**Fig. 3:**
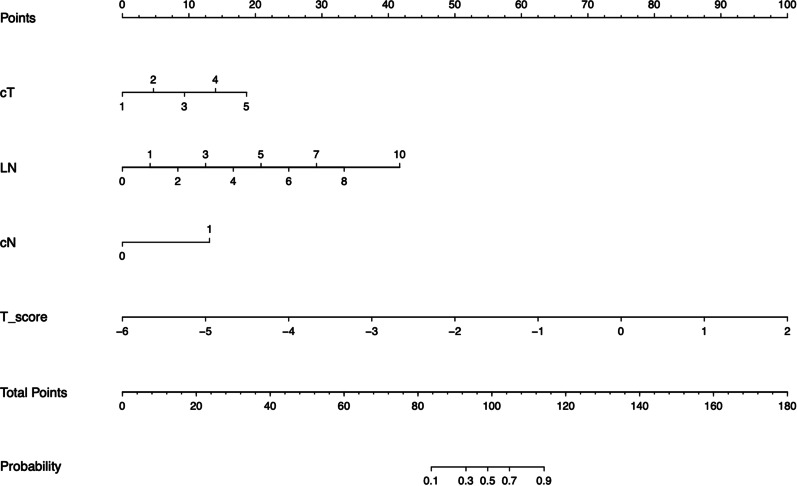
The nomogram of EMVI assessment model. The nomogram incorporated cT, LN, cN, and T-score. cT, the T stage assessed by radiologists based on MRI; LN, the number of visible regional lymph nodes on MRI; cN, the regional lymph node metastasis assessed by radiologists based on MRI; T-score, the texture score calculated by linear combination of the selected features which were weighted by their respective LASSO coefficients

**Fig. 4:**
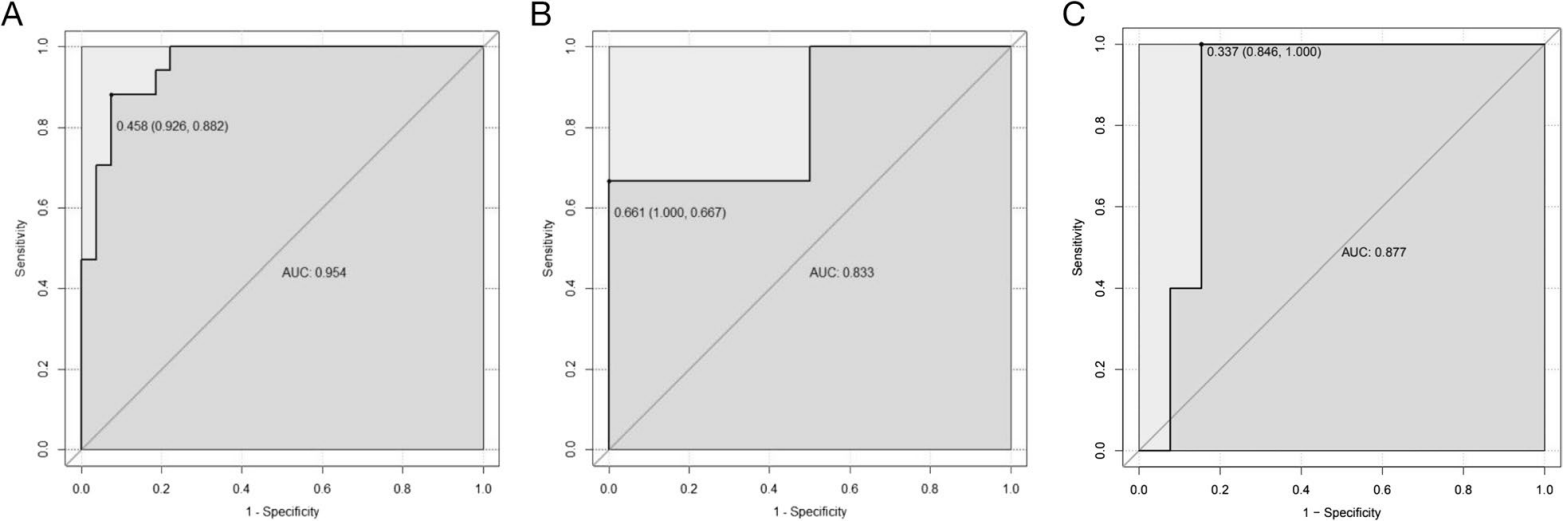
**a** ROC curve of the nomogram in the train cohort. The AUC is 0.954. When the cutoff value is 0.458, the specificity is 0.926 and the sensitivity is 0.882. **b** ROC curve of the nomogram in the validation cohort. The AUC is 0.833. When the cutoff value is 0.661, the specificity is 1.000 and the sensitivity is 0.667. **c** ROC curve of the nomogram in the validation cohort. The AUC is 0.877. When the cutoff value is 0.337, the specificity is 0.846 and the sensitivity is 1.000. ROC, receiver operating characteristic; AUC, area under ROC curve

**Fig. 5:**
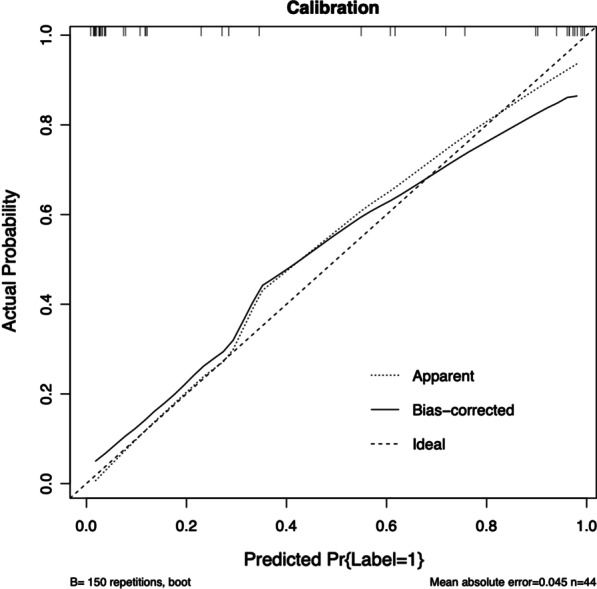
Calibration curve of the nomogram. The Hosmer–Lemeshow test yielded a nonsignificant statistic (*p* = 0.837). Calibration curves describe the model’s calibration in terms of agreement between the predicted probability of EMVI and observed positive proportion of EMVI. The dashed line named Ideal presents perfect performance, the other dashed line named Apparent presents the actual performance of the nomogram, and the solid line named Bias-corrected presents the actual performance of the nomogram after bias-correction

Multivariable logistic regression analysis was used to construct an EMVI assessment model by incorporating the T-score, cT, cN, and LN, which was then presented as a nomogram (Fig. 3). The nomogram with an AUC of 0.954 (95% CI 0.889–0.941), sensitivity of 0.882, and specificity of 0.926 showed better assessment performance than T-score alone (Fig. 4A). A significant difference was found in the ROC curves between the nomogram and T-score using the DeLong test (*Z* = 2.537, *p* = 0.012). The calibration curve for the probability of EMVI showed good agreement between the nomogram-predicted probability of EMVI and the actual EMVI observed (Fig. 5). A nonsignificant statistic (*p* = 0.837) of the Hosmer–Lemeshow test indicated no significant deviation from an ideal fitting.

### Model validation and test

There were 7 cases used to validate the assessment model. The result showed that only one pathological EMVI-positive case was misdiagnosed as EMVI negative, while the EMVI assessments for the remaining 6 cases were all correct. In contrast, all the three EMVI-positive cases were missed and one EMVI-negative case was misdiagnosed as positive by the radiologists. The AUC of the validation cohort was 0.833 with the sensitivity of 0.667 and the specificity of 1.000 (Fig. 4B).

In the test cohort, we saw a similar situation. The model showed a good and stable performance with the AUC of 0.877, the sensitivity of 1.000 and the specificity of 0.846 (Fig. 4C), while all the pathological EMVI-positive cases were missed and all EMVI-positive cases assessed by radiologists were misdiagnosis.

### Clinical practice

**Fig. 6:**
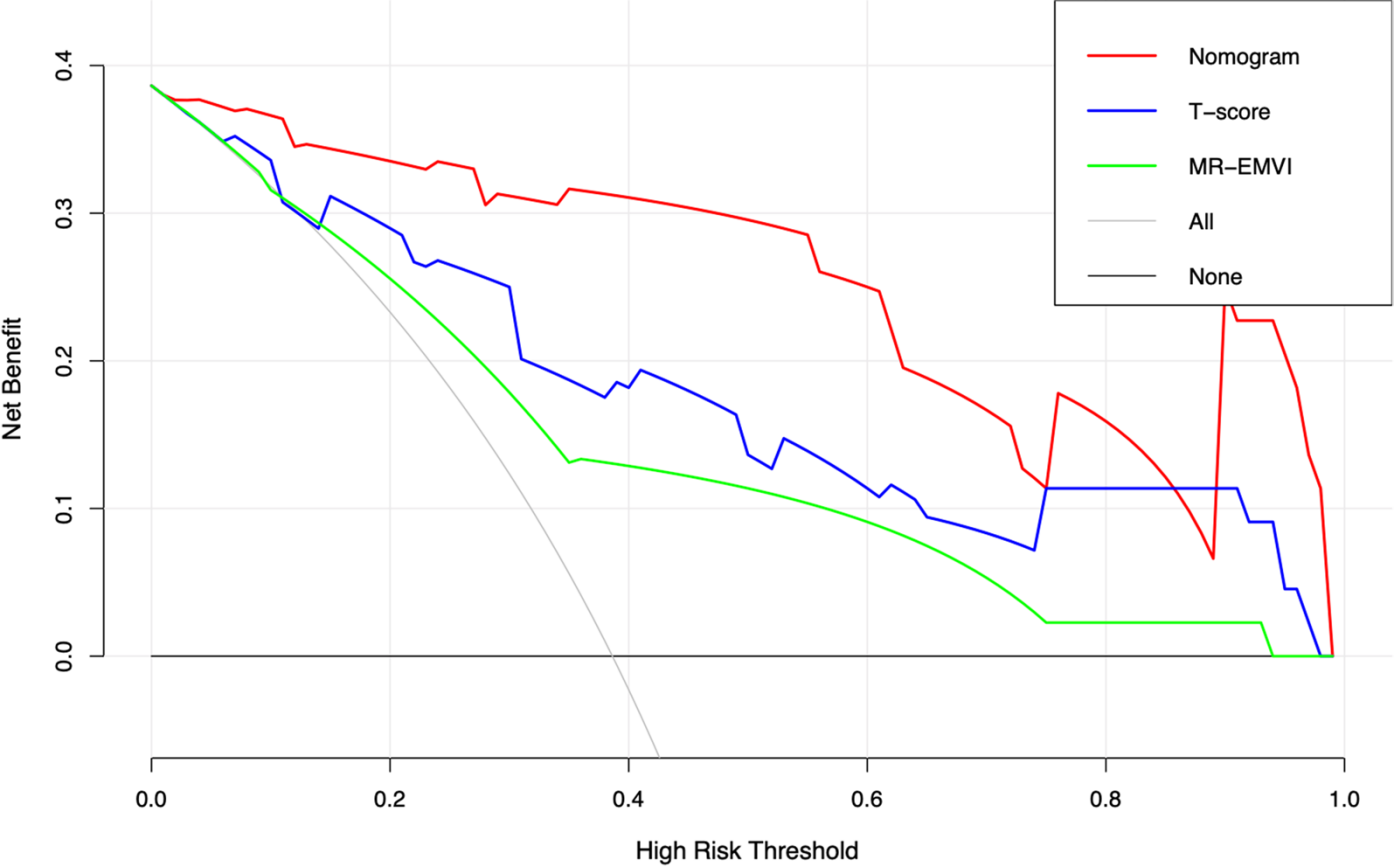
Decision curve analysis for MR-EMVI, T-score and the nomogram of the EMVI assessment model. The black solid horizontal line at the level with Standardized Net Benefit 0 represents the assumption of all rectal cancer patients without EMVI. The grey solid line represents the assumption of all patients with EMVI. The green solid line represents the assumption that patients will be judged EMVI-positive if the positive probability obtained from MR-EMVI is higher than the threshold probability. The blue solid line represents the assumption that patients will be judged EMVI-positive if the positive probability obtained from T-score is higher than the threshold probability. The red solid line represents the assumption that patients will be judged EMVI-positive if the positive probability obtained from the nomogram is higher than the threshold probability. The decision curves showed that when the threshold probability is in a reasonable range, the nomogram provided the greatest net benefit when compared with the treat-all-patients approach, treat-none approach, and the MR-EMVI and T-score for predicting EMVI status of patients with rectal cancer

Among MR-EMVI, T-score, and the assessment model by DCA (Fig. 6), the nomogram of assessment model showed the greatest net benefit in predicting EMVI.

## Discussion

EMVI is a key factor in treatment selection and prognostication of patients with rectal cancer. In a recent study, EMVI was used as an excellent preoperative predictor of poor response to neoadjuvant chemoradiotherapy in patients with locally advanced rectal cancer [27]. An accurate EMVI assessment can provide surgeons with a reliable basis for individualized treatment decisions. According to NCCN guideline [14], MRI is the preferred method for assessing rectal cancer preoperatively. However, the accuracy of EMVI assessment by conventional MRI is limited [14–16]. This study attempted to develop a new EMVI assessment model based on texture analysis to improve preoperative EMVI assessment for patients with rectal cancer.

In our study, we extracted 236 texture features from each patient, including 59 features of *K*^trans^, *K*_ep_, *V*_e_, and *V*_p_, respectively. After feature selection, we found that the key features consisted of three *K*_ep_ features and one *V*_p_ feature. *K*_ep_ features accounted for a significantly greater proportion of key features than other DCE MRI quantitative features. *K*_ep_ is the rate constant of contrast agent escaping from the EES into the plasma compartment, which is affected by unbalanced distribution of blood flow and heterogeneity of the tumor, and may be associated with the vessel invasion. The selected key features, low Kep_Correlation, high Kep_Clustershade, and high Kep_SumEntropy indicate non-homogenous perfusion in rectal cancer. These are also significantly associated with a high risk of EMVI, as seen in the calculation formula for T-score. This may be related to tumor heterogeneity. Vp is the plasma volume fraction, which represents micro-vessel density in rectal cancer. This study found that patients with low Vp_HaraVariance are prone to EMVI, but this may be due to the small sample size. However, there are very limited studies on EMVI and the relationship between Vp and EMVI warrants further investigation.

The items in the conventional MRI assessment and clinical independent risk factors were integrated with the assessment models cT (T stage), cN (regional lymph node metastasis), and LN (number of visible regional lymph nodes), as assessed by radiologists based on MRI. This is consistent with some previous studies, which showed that high T and N stages were risk factors for EMVI [20, 28]. Interestingly, LN showed a more significant correlation with EMVI than cN. A recent study explained that increasing levels of interleukin 6 receptor (IL6R) and plasminogen activator inhibitor 1 (PAI1) promote colorectal cancer development, progression, and metastasis and participate in inflammation in mice [29]. Thus, LN may facilitate the assessment of EMVI because it reflects not only lymph node metastasis, but also inflammation.

According to previous studies, the main problem of conventional MRI EMVI assessment is low and unstable sensitivity [14, 15]. In this study, 17 patients in the train cohort, 3 patients in the validation cohort and 5 patients in the test cohort had EMVI confirmed by pathological diagnosis, but only four patients were seen to be EMVI-positive by radiologists on conventional MRI. The low sensitivity of 23.53% in the train cohort is similar to that of 28.2% in Sohn et al.’s study [16]. The pathological diagnostic criteria of EMVI in the both studies were pathologic lymphovascular invasion without specifying the intra- or extra-mural invasion, which may explain the low sensitivity in Sohn's study. Reviewing the cases in our study, we found most of them were at early stage of EMVI because those obvious ones ought to be treated with neoadjuvant chemoradiotherapy, which probably was the reason why the accuracy of radiologists was so low.

In a recent study, the radiomics of DCE MRI showed better diagnostic performance with an AUC of 0.872 in the training cohort and 0.812 in the validation cohort [26] than clinical features. However, this study used postoperative histopathological information to assess EMVI, and their best radiomics nomogram contained two histopathological items: histological grade and histologic tumor stage. This means that this method was invasive. Compared with their radiomics model based on DCE MRI, T-score also demonstrated a markedly better classification performance than the radiologists’ assessment of EMVI based on conventional MRI with an AUC of 0.797. This indicates that the texture analysis had an incremental value in assessing EMVI of rectal cancer. Moreover, our nomogram, the integration of T-score, conventional MRI assessment, and clinical independent risk factors showed a better performance than other previously reported methods with better AUCs of 0.954 in train cohort, 0.833 in the validation cohort and 0.877 in the test cohort, respectively. In addition to this, its components are all noninvasive.

There were several limitations in this study. First, because this was a single-center study and the sample size was small, the data could not represent the entire rectal cancer population, and the EMVI assessment model needs further investigation using a larger dataset with external validation to check for the existence of overfitting and evaluate its robustness and reproducibility. Second, EMVI is a focal feature along the surface of the tumor/adjacent or in the peripheral tissues; however, in this study, the ROIs only covered the whole tumor, possibly causing a loss of information about EMVI in the peripheral tissues. Third, because this study did not include the patients who underwent neoadjuvant chemoradiotherapy, the data were not so comprehensive that reflected all rectal cancer patients. The assessment of EMVI in rectal cancer patients with neoadjuvant CRT will  be discussed in our further research (Additional file 1).

## Conclusions

In conclusion, we developed a novel assessment model for EMVI in rectal cancer by texture analysis based on DCE MRI. The EMVI assessment model showed better performance in preoperative assessment of EMVI. This assessment model shows potential for improving MRI-based EMVI assessment, especially in patients with early stage of EMVI, and provide a reliable basis for individualized treatment decisions.

## Supplementary Information

**Additional file 1. Appendix A1.** Conventional MR protocol. **Appendix A2.** The key texture features. **Figure S1.** Recruitment pathway for patients in this study.

## Data Availability

The datasets used and/or analyzed during the current study are available from the corresponding author on reasonable request.

## Abbreviations

AIC: Akaike information criterion
AIF: Arterial input function
ANOVA: Analysis of variance
AUC: Area under the curve
BMI: Body mass index (BMI)
CA19-9: Carbohydrate antigen 19-9
CEA: Carcinoembryonic antigen
cN: MRI-based regional lymph node metastasis assessment
CRM: Circumferential resection margin
cT: MRI-based T staging
CT: Computed tomography
DCA: Decision curve analysis
DCE: Dynamic contrast enhancement
EES: Extracellular extravascular space
EMVI: Extramural vascular invasion
EUS: Endoscopic ultrasound
GLCM: Grey-level co-occurrence matrix
ICC: Interclass correlation coefficient
LASSO: Least absolute shrinkage and selection operator
LN: Number of visible regional lymph nodes on MR images
MR-EMVI: MRI-based EMVI assessment
MRF: Mesorectal fascia
MRI: Magnetic resonance imaging
nCRT: Neoadjuvant chemoradiotherapy
RLM: Grey-level run-length matrix
ROC: Receiver operating characteristic curve
ROI: Region of interest
TA: Texture analysis
