# Supplementary material for: Value of texture analysis based on dynamic contrast-enhanced magnetic resonance imaging in preoperative assessment of extramural venous invasion in rectal cancer
Source: Insights Imaging. 2022 Nov 22;13:179. doi: 10.1186/s13244-022-01316-2 (PMC9684393; doi:10.1186/s13244-022-01316-2)
Supplement: Supplementary file 1 — Additional file 1. Appendix A1. Conventional MR protocol. Appendix A2. The key texture features. Figure S1. Recruitment pathway for patients in this study. [file 13244_2022_1316_MOESM1_ESM.docx]

**Appendix for “Value of texture analysis based on dynamic contrast-enhanced magnetic resonance imaging in preoperative assessment of extramural venous invasion in rectal cancer”**

# Appendix A1. Conventional MR protocol

All patients underwent a conventional rectal MRI protocol including high-resolution T2-weighted imaging (HR-T2WI), diffusion weighted imaging (DWI), axial T1-weighted imaging (T1WI), axial, coronal and sagittal T2-weighted imaging (T2WI) and contrast enhanced MR imaging (CE-MRI). Scan parameters were as follows:

**HR-T2WI:** repetition time (TR) = 3580 ms; echo time (TE) = 95 ms; field-of-view (FOV) = 180 × 180 mm2; matrix = 320 × 256; slice thickness = 3.0 mm; spacing = 0.3 mm.

**DWI:** performed by single-shot echo-planar imaging (EPI) with 2 b-factors (0 and 800 s/mm2); TR = 2900 ms; TE = 83 ms; FOV = 300 × 300 mm^2^; matrix = 130 × 130; slice thickness = 5.0 mm, and spacing = 1.0 mm.

**T1WI:** TR = 610 ms; TE = 7.30 ms; FOV = 240 × 240 mm^2^; matrix = 256 × 205; slice thickness = 5.0 mm, and spacing = 1.0 mm.

**T2WI:** Axial: TR = 3500 ms; TE = 80 ms; FOV = 240 × 240 mm^2^; matrix = 320 × 256; slice thickness = 5.0 mm, and spacing = 1.0 mm. Coronal: TR = 3210 ms; TE = 81 ms; FOV = 300 × 300 mm^2^; matrix = 320 × 256; slice thickness = 5.0 mm, and spacing = 1.0 mm. Sagittal: TR = 3560 ms; TE = 84 ms; FOV = 260 × 260 mm^2^; matrix = 320 × 256; slice thickness = 4.0 mm, and spacing = 0.8 mm.

**CE-MRI:** performed after DCE-MRI with the flip angle of 10°, including a coronal, a sagittal and an axial imaging. Coronal: TR = 3.42 ms; TE = 1.35 ms; Average = 1; FOV = 320 × 320 mm^2^; matrix = 288 × 207; slice thickness = 2.0 mm. Sagittal: TR = 3.42 ms; TE = 1.35 ms; Average = 1; FOV = 320 × 320 mm^2^; matrix = 288 × 207; slice thickness = 2.0 mm. Axial: TR = 4.93 ms; TE = 2.39 ms; FOV = 350 × 280 mm^2^; matrix = 320 × 220; slice thickness = 2.0 mm.

# Appendix A2. The key texture features

The T score is consisted of the 4 optimal key texture features, which were named ***Kep_Correlation***, ***Kep_Clustershade***, ***Kep_SumEntropy*** and ***Vp_HaraVariance***. The first three features were extracted from K_ep_ maps and the last feature was from V_p_ maps.

***Correlation*** measures the linear dependency of grey levels of neighboring pixels, in other words, it measures the similarity of the grey levels in neighboring pixels, tells how correlated a pixel is to its neighbor over the whole image.

Cluster analysis is the task of grouping a set of objects in such a way that objects in the same group (cluster) are more similar (in some sense or another) to each other than to those in other groups (clusters). ***Cluster Shade*** is grouped similar view samples according to their position.

***SumEntropy*** and ***HaraVariance*** are both Haralick features, which describes image texture statistically. ***SumEntropy*** is a measure of randomness of the distribution of the coefficients values over the intensity levels, having a low value in a simple image while a high value in a complex image. ***HaraVariance*** is the average of the squared differences from ***Mean***. ***Mean*** measures the average value of the intensity values.

# Appendix A3. Supplementary tables and figures

80 patients with rectal cancer were collected from May, 2019 to December, 2019 according with inclusion criteria

69 patients included for segmentation and analysis

3 patients excluded with artifacts

3 patients excluded because displayed incompletely on DCE images

5 patients excluded with presenting other malignancy

**Figure S1.** Recruitment pathway for patients in this study
